# Drug Combinations: A New Strategy to Extend Drug Repurposing and Epithelial-Mesenchymal Transition in Breast and Colon Cancer Cells

**DOI:** 10.3390/biom12020190

**Published:** 2022-01-23

**Authors:** Diana Duarte, Alexandra Rêma, Irina Amorim, Nuno Vale

**Affiliations:** 1 OncoPharma Research Group, Center for Health Technology and Services Research (CINTESIS), Rua Doutor Plácido da Costa, 4200-450 Porto, Portugal; dianaduarte29@gmail.com; 2 Faculty of Pharmacy, University of Porto, Rua Jorge Viterbo Ferreira, 228, 4050-313 Porto, Portugal; 3 Department of Pathology and Molecular Immunology, Institute of Biomedical Sciences Abel Salazar (ICBAS), University of Porto, 4050-313 Porto, Portugal; alexandra.rema@gmail.com (A.R.); iamorim@ipatimup.pt (I.A.); 4 Institute of Pathology and Molecular Immunology, University of Porto (IPATIMUP), 4200-465 Porto, Portugal; 5 i3S-Instituto de Investigação e Inovação em Saúde, University of Porto, 4200-135 Porto, Portugal; 6 Department of Community Medicine, Health Information and Decision (MEDCIDS), Faculty of Medicine, University of Porto, Alameda Professor Hernâni Monteiro, 4200-319 Porto, Portugal

**Keywords:** drug synergism, drug repurposing, CNS drugs, combination therapy, epithelial-mesenchymal transition

## Abstract

Despite the progressive research and recent advances in drug therapy to treat solid tumours, the number of cases and deaths in patients with cancer is still a major health problem. Drug repurposing coupled to drug combination strategies has been gaining interest among the scientific community. Recently, our group proposed novel drug combinations for breast and colon cancer using repurposed drugs from different classes (antimalarial and central nervous system (CNS)) and chemotherapeutic agents such as 5-fluorouracil (5-FU), paclitaxel (PTX), and found promising results. Here, we proposed a novel drug combination using different CNS drugs and doxorubicin (DOX), an antineoplastic used in breast cancer therapy, and studied their anticancer potential in MCF-7 breast cancer cells. Cells were treated with each drug alone and combined with increasing concentrations of DOX and cell viability was evaluated by MTT and SRB assays. Studies were also complemented with morphological evaluation. Assessment of drug interaction was performed using the CompuSyn and SynergyFinder software. We also compiled our previously studied drug pairs and selected the most promising ones for evaluation of the expression of EMT biomarkers (E-cadherin, P-cadherin, vimentin, and β-catenin) by immunohistochemistry (IHC) to assess if these drug combinations affect the expression of these proteins and eventually revert EMT. These results demonstrate that combination of DOX plus fluoxetine, benztropine, and thioridazine at their IC_50_ can improve the anticancer effect of DOX but to a lesser degree than when combined with PTX (previous results), resulting in most of the drug interactions being antagonist or additive. This suggests that the choice of the antineoplastic drug influences the success of the drug combination. Collectively, these results also allow us to conclude that antimalarial drugs as repurposed drugs have enhanced effects in MCF-7 breast cancer cells, while combination with CNS drugs seems to be more effective in HT-29 colon cancer cells. The IHC results demonstrate that combination treatments increase E-cadherin expression while reducing P-cadherin, vimentin, and β-catenin, suggesting that these treatments could induce EMT reversal. Taken together, these results could provide promising approaches to the design of novel drug combinations to treat breast and colon cancer patients.

## 1. Introduction

Epithelial-mesenchymal transition (EMT) is a complex cellular process where cells lose their epithelial features and acquire mesenchymal characteristics. This change gives cells mobility and consequently the ability to migrate from the primary site [1]. Although EMT is normal in embryonic stages [2], in adults, EMT is usually related to the healing process or cancer metastasis [3]. EMT also explains the aggressive phenotype and malignancy of different types of cancer [4]. Several studies suggest that EMT is linked to cancer progression [5,6,7]. 

Different biomarkers can be used in EMT studies through evaluation of their level of expression, distribution, and changes of function, which help to characterize the state in which cancer cells are. These biomarkers include growth factors, such as TFG-β and Wnts, transcription factors such as SNAIL and TWIST, adhesion molecules such as cadherins, and molecules present in the cytoskeleton as vimentin [8]. 

TGF-β regulates cell proliferation and is one of the main regulators of EMT: it is a suppressor of epithelial cells and leads to the loss of epithelial proteins such as E-cadherin while increasing the expression of mesenchymal biomarkers such as vimentin [9]. Snail, a transcription factor induced by TGF-β, also increases the expression of mesenchymal phenotype while decreasing epithelial proteins such as E-cadherin [10]. Twist, another transcription factor, regulates the shift from E-cadherin to a less adhesive N-cadherin, a characteristic cadherin in mesenchymal cells [1]. P-cadherin is usually co-expressed with E-cadherin, and its expression is increased in some disease states, namely genetic disorders or cancer progression [11]. Another marker of EMT is the β-catenin expression, which is overexpressed in more advanced cases with worse prognoses [12]. Vimentin is a cytoskeleton protein, typically overexpressed in mesenchymal cells, and its increased expression has been observed in several carcinomas such as breast and colon, associated with malignancy and metastasis [13,14,15]. 

In this study, our group hypothesized that the combination of antineoplastic agents and repurposed drugs could help reverse the EMT in breast and colon cancer cells. Drug repurposing is a strategy that has been gaining visibility in cancer therapy research as an alternative to the traditional process of new drug development. This strategy tries to find new uses for drugs that are already approved by the FDA besides their original indication, allowing the reduction of the costs and time associated with the development of new drugs. As these drugs are already available in the market, they have pharmacological and toxicological profiles well-established, and their approval for novel indications can be easier compared to new drugs. Several studies have reported the potential use of repurposed drugs in cancer therapy [16,17,18]. The drug combination is a strategy that consists of the administration of a cocktail of two or more drugs [19] and allows overcoming the intra- and intertumoral heterogeneity that is usually related to tumour progression, drug resistance, and lack of efficacy [20]. Several studies have demonstrated that drug combination is more effective in reducing cancer cell proliferation and viability than monotherapy [21,22,23,24,25,26], especially if synergism is achieved, allowing the reduction of therapeutic dosage and, consequently, side effects. Our group has some experience with combination models for cancer therapy, and, in previous works, we already combined repurposed drugs from different classes with antineoplastic agents and have found promising drug pairs for breast and colon cancer therapy [27,28]. Here, we evaluated a novel combination of different central nervous system (CNS) agents with doxorubicin (DOX), an antineoplastic agent commonly used in breast cancer therapy, using the combination model that was previously described [28]. The repurposing of CNS drugs has already been explored with different studies reporting the efficacy of these agents to reduce the viability of cancer cells. Different CNS drugs have already demonstrated some repurposing potential, such as imipramine [29,30,31,32,33], phenothiazines [34,35,36], trifluoperazine [37,38], pimozide [39], and valproic acid [40,41,42]. 

Previously, we have already evaluated the combination of CNS drugs with paclitaxel (PTX) and 5-fluorouracil (5-FU), two antineoplastic agents used in MCF-7 breast and HT-29 colon cancer cells, respectively, and found that some drug pairs could synergistically decrease the proliferation and viability of cancer cells [27]. This work is a follow-up to our previously published articles [27,28], and we have divided our manuscript into two main parts: first, and based on our previous findings, we hypothesized that CNS drugs could also help increase the cytotoxicity of DOX, in combination therapies, in MCF-7 breast cancer cells. We then compiled our previous results obtained in HT-29 colon and MCF-7 breast cancer cells with these novel results. In the second part, and to understand how the drugs act in combination and if EMT is involved, we evaluated our most promising drug pairs since the beginning of our project, regarding the use of repurposed drugs (antimalarial and CNS agents) combined with antineoplastic agents in two different cell lines (HT-29 and MCF-7 cells), for the expression of EMT biomarkers, such as E-cadherin, P-cadherin, vimentin, and β-catenin, to assess if all drug pairs would affect EMT the same way, using different classes of repurposed drugs and cell lines. MCF-7 breast and HT-29 colon cancer cells are widely used cell lines, with epithelial characteristics. Some studies suggest they can acquire mesenchymal features under malignancy and drug resistance.

We have found that CNS drugs combined with DOX are less effective than when combined with PTX in the reduction of MCF-7 cell viability, with almost all drug pairs being additive or antagonists. We also found that EMT is indeed affected by the treatments, with a reversal of EMT in cells treated with the most promising combinations, resulting in increased expression of E-cadherin and P-cadherin, with lower expression of mesenchymal markers such as vimentin and β-catenin, which results in a less aggressive phenotype and drug resistance of these cells. 

## 2. Materials and Methods

### 2.1. Materials and Reagents

Cell culture reagents such as McCoy’s 5A Modified Medium, Dulbecco’s Modified Eagle Medium (DMEM), fetal bovine serum (FBS), and a mixture of pen/strep solution were obtained from Millipore Sigma (Merck KGaA, Darmstadt, Germany). Other cell culture reagents were brought from Gibco (Thermo Fisher Scientific, Inc, Waltham, MA, USA). Fluphenazine (cat. no. F4765), latrepirdine (cat. no. D6196), 5-FU (cat. no. F6627), Thiazolyl Blue Tetrazolium Bromide (MTT, cat. no. M5655), Bovine Serum Albumin (BSA, cat. no. A8531), sulforhodamine B (SRB, cat. no. S1402), Entellan mounting medium (cat. no. 107960), and (3-Aminopropyl)triethoxysilane (silane, cat. no. A3648) were obtained from Sigma-Aldrich (Merck KGaA, Darmstadt, Germany). Benztropine (cat. no. 16214), sertraline (cat. no. 14839), thioridazine (cat. no. 14400), fluoxetine (cat. no. 14418), artesunate (cat. no. 11817), and doxorubicin (cat. no. 15007) were acquired from Cayman Chemical (Ann Arbor, MI, USA). Chloroquine (cat. no. C6628) was purchased from Santa Cruz Biotechnology (Dallas, TX, USA). PTX (cat. no. 1097) was obtained from Tocris Bioscience (Bristol, UK). Perampanel was purchased from Eisai Co., Ltd. (Tokyo, Japan). Novolink Max-Polymer detection system was bought from Novocastra (Newcastle, UK). 

### 2.2. Evaluation of the Cytotoxic Effect of CNS Drugs Combined with DOX in MCF-7 Cells

#### 2.2.1. Cell Line and Cell Culture

This work was carried out using an MCF-7 cell line (ATCC, American Type Culture Collection, Manassas, VA, USA). MCF-7 cells were maintained in DMEM cell culture medium supplemented with 10% FBS and 1% of a mixture of penicillin G and streptomycin (1000 U/mL; 10 mg/mL) at 37 °C in a humidified atmosphere with 95% air and 5% CO_2_. Cells were cultured in monolayer in T25 cm^2^ flasks and cell media was changed two times a week. Cells were subcultured once a week using a solution of 0.25% trypsin-EDTA when confluence reached 70–80%. 

For the experiments, and before drug exposure, MCF-7 cells were plated in 96-well plates using a density of 5000 cells per well, using a final volume of 200 µL. Cells were allowed to adhere overnight, and, after 24 h, cell media were aspirated and replaced with 200 µL of drug-containing media for 48 h. After this time, different cell-based assays (MTT and SRB assays) were performed to evaluate the cytotoxic effect of each treatment in the cell viability and protein synthesis rate of these cells.

#### 2.2.2. Cell Treatment

After single drug treatment, the half-maximal inhibitory concentration (IC_50_) value was determined in MCF-7 cells. For that, cells were treated with each drug in concentrations between 0.1 and 100 µM. This value was calculated as the concentration causing 50% cell growth inhibition compared to control cells. For the combined treatments, cells were incubated with DOX (Drug A) and different CNS agents (Drug B) simultaneously. Drugs that presented an IC_50_ under 25 µM were selected for testing combined with DOX. The concentration of both drugs in combination were variable and in a fixed ratio of the IC_50_ values for each drug (0.25, 0.5, 1, 2, and 4 times the IC_50_). Cells treated with vehicle (0.1% DMSO) were used as control.

#### 2.2.3. Cell-Based Assays

Two cell-based assays were used to determine the antitumor effect of each drug alone and in combination on MCF-7 cells: MTT and SRB assays. For the MTT assay, after cell seeding and treatment, cell media was aspirated and replaced with 100 μL per well of MTT solution (0.5 mg/mL in phosphate-buffered saline (PBS)). Then, cells were maintained at 37 °C for 3 h in a light-protected manner. At the end of this time, the MTT solution was aspirated, and 100 μL/well of DMSO was added to dissolve the formazan crystals. The measure of absorbance at 570 nm was performed using an automated microplate reader (Tecan Infinite M200, Tecan Group Ltd, Männedorf, Switzerland). 

Regarding the SRB protocol, after cell seeding and treatment, cells were fixed using an ice-cold 10% trichloroacetic acid solution for 30 min and incubated with a 0.4% SRB solution for 1 h at room temperature. To remove the excess dye, plates were washed three times with tap water and allowed to dry. To quantify protein-bound dye, 200 µL of 10 mM Tris base solution was added to each well, and absorbance readings were performed at 510 nm using an automated microplate reader (Tecan Infinite M200, Tecan Group Ltd, Männedorf, Switzerland). Each experiment was repeated three times, using triplicates. 

#### 2.2.4. Cell Morphology Visualization

After drug treatment, the morphological features of MCF-7 cells were captured using a Leica DMI 6000B microscope coupled to a Leica DFC350 FX camera. Images were then analyzed using the Leica LAS X imaging software (v3.7.4).

#### 2.2.5. Data Analysis

To obtain the concentration-response curves, we analyzed the results by nonlinear regression using the GraphPad Prism 8 software (GraphPad Software Inc., San Diego, CA, USA). For that, we normalized the viability of cells treated with each compound to the viability of control cells, and the cell viability fractions were plotted vs. drug concentrations on the logarithmic scale. 

#### 2.2.6. Evaluation of Synergism Using CompuSyn and SynergyFinder Software 

After drug combination treatment, and using the MTT results, the Combination Index (CI) was calculated using the CompuSyn Software (ComboSyn, Inc., New York, NY, USA) to investigate the drug interaction nature in each combination using DOX and CNS agents. This parameter was first introduced by Chou and Talalay [43] and is based on the unified theory proposed by these authors. This model assumes that drugs act through entirely different mechanisms [44]. Representation of results was performed by plotting CI on the *y*-axis and the effect level (Fa) on the *x*-axis. The CI value is indicative of the pharmacological interactions between the two drugs in a combination, representing synergism, additivity, or antagonism if its value is under, equal, or above 1, respectively. Drug interactions were also quantified by another reference model, the Bliss model, using the software SynergyFinder [45]. In this model, positive or negative values represent synergy and antagonism, respectively. 

#### 2.2.7. Statistical Analysis

Statistical analysis was performed using the results obtained in three independent experiences, in triplicates. All results are expressed as mean ± SEM for n experiments performed. Differences between treatment groups and corresponding untreated control cells were tested using Student’s *t*-test and one-way ANOVA test in GraphPad Prism 7 (San Diego, CA, USA). Statistical significance was considered when *p* values < 0.05.

### 2.3. Evaluation of EMT Biomarkers in MCF-7 and HT-29 Cells Treated with Repurposed Drugs Combined with Antineoplastic Agents

#### 2.3.1. Cell Line and Cell Culture 

This protocol was carried out using two different cell lines: MCF-7 and HT-29 cell lines (ATCC, American Type Culture Collection, Manassas, VA, USA). MCF-7 and HT-29 cells were maintained in DMEM and McCoy’s cell culture medium, respectively, in the conditions described in Section 2.2.1. After reaching 70–80% confluence, cells were prepared for immunohistochemistry. 

#### 2.3.2. Preparation of Cells for Immunohistochemistry (IHC)

We first adapted the protocol for IHC for 24 well plates, using adherent cells. Sterile 13 mm coverslips were placed in 24-well plates and coated with silane 2% in acetone for 1 h at room temperature. Before cell seeding, silane coverslips were washed with PBS and allowed to dry for 2 h. Next, MCF-7 and HT-29 cells were seeded with a density of 12,500 and 37,500 cells/well, respectively, and incubated for 24 h at 37 °C. After that period, cells were incubated with single and combination treatments for 48 h. Cells treated with vehicle (0.1% DMSO) were used as control. Cells were then washed with PBS and incubated with a solution of paraformaldehyde (PFA) 4% in PBS for 15 min at room temperature, for cell fixation. Next, cells were washed three times with PBS-0.1% Tween 20 and maintained in PBS at 4 °C until the beginning of the IHC protocol. 

#### 2.3.3. Immunohistochemistry Protocol

For the IHC protocol, a Novolink Max-Polymer detection system was used, according to the manufacturer’s instructions. The detailed protocol is described in Supplementary Figure S1. Primary antibodies included anti-E-cadherin (4A2C7; 1:1000 in BSA 5%; Invitrogen, MA, USA), anti-P-cadherin (56/P-Cadherin; 1:150 in BSA 5%; BD Biosciences, NJ, USA), anti-β-catenin (CAT-5H10; 1:900 in BSA 5%; Invitrogen, MA, USA), and anti-vimentin (V9; 1:1000 in BSA 5%; Dako, Denmark). The negative control refers to cells that were not incubated with the primary antibody. After IHC protocol, each coverslip was removed from the 24-well plate using a small pair of broad-tipped forceps and rinsed in water, counterstained in hematoxylin for 30 s, washed again for 5 to 10 min, dehydrated (at increasing concentrations of ethanol), and diaphanized in xylene, and the slides were mounted using Entellan. Each slide was analyzed using a Nikon Eclipse E600 microscope coupled to a digital camera (Nikon Digital Sight DS-Fi2, Tokyo, Japan). Images were then captured using the Imaging Software NIS-Elements AR Version 4.30.0 (Nikon, Tokyo, Japan).

## 3. Results

### 3.1. Evaluation of the Cytotoxic Effect of CNS Drugs Combined with DOX in MCF-7 Cells 

#### 3.1.1. Single Treatment of MCF-7 Breast Cancer Cells with CNS Drugs

In our previous results [27], we have found that combination of CNS drugs with two antineoplastic agents (5-FU in HT-29 colon cancer cells and with PTX in MCF-7 breast cancer cells) resulted in enhanced anticancer activity by synergic interactions in these cell lines when compared to the drugs alone. Based on these findings, in this study, we tried to evaluate if the combination of those CNS agents with another antineoplastic agent commonly used in breast cancer therapy, DOX, would also result in promising results. 

**Figure 1:**
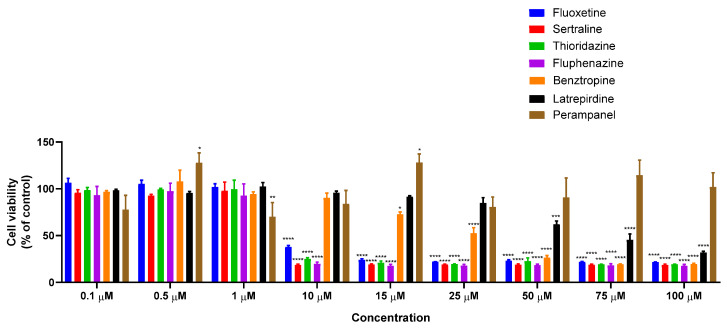
Viability of MCF-7 breast cancer cells incubated with different CNS drugs alone. Cultured cells were seeded in 96-well plates and treated with each drug alone (0.1–100 μM) for 48 h. Cells treated with vehicle (0.1% DMSO) were used as control. Cell viability was determined after each treatment by MTT assay. Each bar represents the mean ± SEM relative to the control cells. * Statistically significant vs. control at *p* < 0.05. ** Statistically significant vs. control at *p* < 0.01. *** Statistically significant vs. control at *p* < 0.001. **** Statistically significant vs. control at *p* < 0.0001.

To determine the antitumor potential of the combination of DOX and CNS drugs, we first treated MCF-7 cells with increasing concentrations of each CNS drug alone (0.1–100 µM) for 48 h. The cell viability results showed that treatment with fluoxetine, sertraline, thioridazine, fluphenazine, benztropine, and latrepirdine alone resulted in concentration-dependent growth inhibition (Figure 1). Curiously, perampanel demonstrated better anticancer activity for lower concentrations than in higher concentrations.

Compared to our previous results, all selected CNS drugs resulted in higher IC_50_ values in MCF-7 cells than HT-29 cells (Table 1), except for sertraline, revealing a decreased anticancer potential of this drug class for breast cancer therapy. In Table 1, our previous results obtained with another class of repurposed drugs (antimalarial) for both cell lines are also represented to help the reader understand the choice of the drug pairs evaluated for EMT biomarkers in Section 3.2. The CNS agents that presented an IC_50_ under 25 µM were selected for combination with DOX and evaluated by MTT and SRB assays, as well as morphological analysis. 

#### 3.1.2. Co-Treatment of MCF-7 Breast Cancer Cells with DOX and CNS Drugs

**Figure 2:**
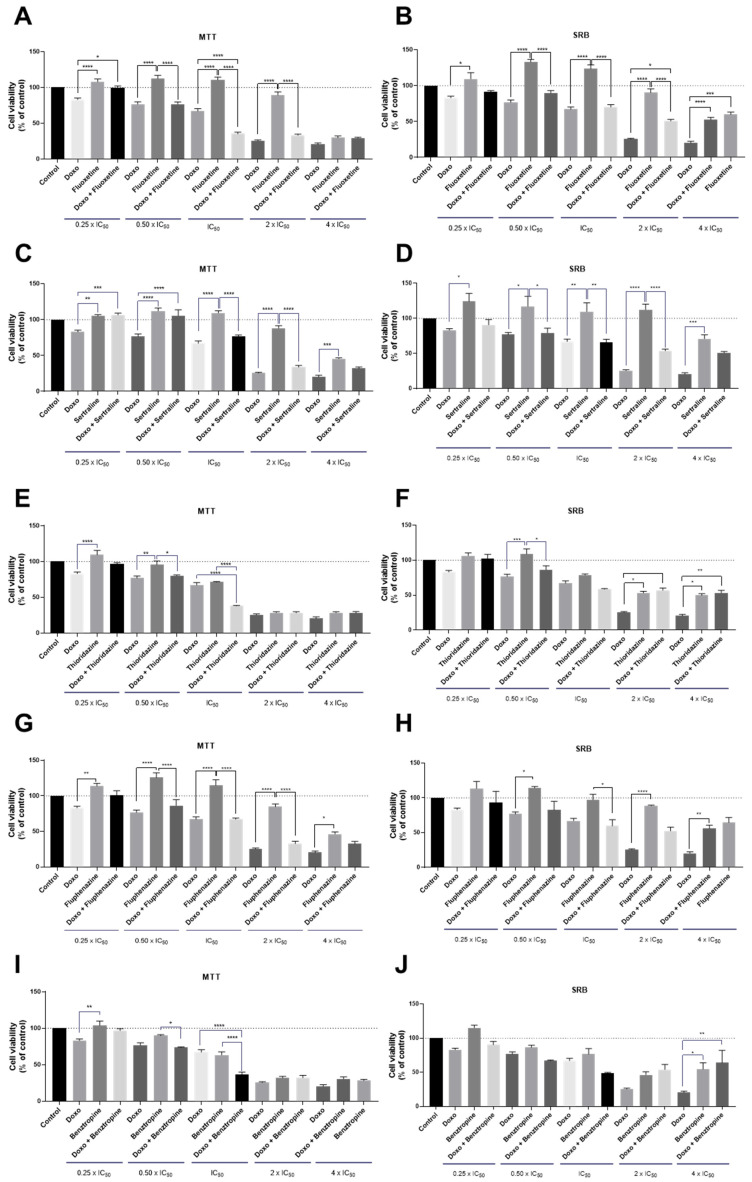
Cell viability of MCF-7 breast cancer cells treated with the combination of different CNS drugs and DOX, by MTT (left) and SRB assays (right). Cultured cells were seeded in 96-well plates and treated with concentrations of each drug of 0.25, 0.5, 1, 2, and 4 times their IC_50_ for 48 h. Cells treated with vehicle (0.1% DMSO) were used as the control. Cell viabilities were determined after the final treatment by MTT and SRB assays. The drugs were co-administered in combination. (**A**) The effect of DOX + fluoxetine on cell viability and (**B**) cell protein synthesis. (**C**) The effect of DOX + sertraline on cell viability and (**D**) cell protein synthesis. (**E**) The effect of DOX + thioridazine on cell viability and (**F**) cell protein synthesis. (**G**) The effect of DOX + fluphenazine on cell viability and (**H**) cell protein synthesis. (**I**) The effect of DOX plus benztropine on cell viability and (**J**) cell protein synthesis. Each point represents the mean ± SEM relative to the control cells. * Statistically significant vs. control at *p* < 0.05. ** Statistically significant vs. control at *p* < 0.01. *** Statistically significant vs. control at *p* < 0.001. **** Statistically significant vs. control at *p* < 0.0001.

After determining the anticancer potential of each CNS drug in MCF-7 breast cancer cells, we selected the drugs with IC_50_ under 25 µM and combined them with increasing concentrations of DOX for 48 h. The obtained results are supported by two cell-based assays: MTT and SRB. The combination design of these experiences was the same developed by us in our previous studies [28]. Co-treatment of MCF-7 cells with fluoxetine and DOX at concentrations of IC_50_ significantly enhanced the cytotoxicity, resulting in a decrease in cell viability compared to the treatments with DOX and fluoxetine alone (Figure 2A). No significant differences in the cellular protein content were observed in cells co-treated with fluoxetine and DOX (Figure 2B). The combination of sertraline and DOX proved to be less effective in promoting cytotoxic effects than each drug alone, for all concentrations tested, both by MTT and SRB assays (Figure 2C,D). Co-treatment of thioridazine with DOX proved to be effective and significantly different from thioridazine and DOX alone at the concentration of IC_50_ of each drug (Figure 2E). The combination of fluphenazine proved not to be advantageous over each drug alone, both by MTT and SRB (Figure 2G,H). Benztropine combined with DOX at the concentration of IC_50_ resulted in significant cell viability reduction compared to DOX and benztropine alone (Figure 2I). Compared to our previous results [27], these findings demonstrate that the combination of CNS agents with PTX has more anticancer potential than with DOX, with more cytotoxic effects for MCF-7 cancer cells. Nevertheless, these results demonstrate that fluoxetine, thioridazine, and benztropine combined with DOX at the concentrations of IC_50_ are promising drug pairs to further evaluate mechanistically and for dose adjustment. Morphologically, all treatments resulted in a lower number of viable cells compared to the control well and treatment with DOX alone (Figure S2). Linear regression analysis of the results obtained by MTT and SRB assays is represented in Figure S3. 

#### 3.1.3. Evaluation of Synergism in MCF-7 Cells Co-Treated with DOX and Different CNS Drugs

**Figure 3:**
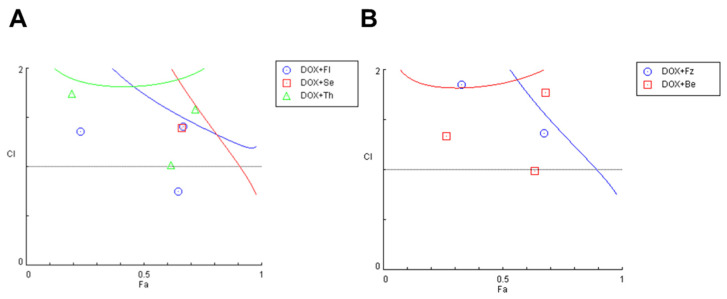
Fa-CI plot of combined treatments of DOX and different CNS drugs on MCF-7 breast cancer cells. For simplification, results were split into two different plots. (**A**) Fa-CI plot of DOX+fluoxetine (blue), DOX+sertraline (red) and DOX+Thioridazine (green). (**B**) Fa-CI plot of DOX+fluphenazine (blue) and DOX+benztropine (red). CI was determined using CompuSyn software. CI < 1, =1, and >1 indicate synergistic, additive, and antagonistic effects, respectively. Fl: fluoxetine; Se: sertraline; Th: thioridazine; Fz: fluphenazine; Be: benztropine.

After finding the most promising drug pairs, we tried to understand if the improvements observed in the co-treatments were due to synergistic interactions between the drugs. To do so, we calculated the CI values for each drug pair using the CompuSyn Software. As shown in Figure 3, the CI values of DOX and CNS drugs in combination were mostly higher than one, except for one pair of DOX + fluoxetine, suggesting that the growth inhibitory effect of these compounds in combination was mostly additive or antagonistic in MCF-7 cells.

Compared to our previously published results [27], we can verify that CNS drugs work better in HT-29 colon cancer cells, both alone and combined with 5-FU, than in MCF-7 cells. When combined with PTX in MCF-7 cells, combination with CNS drugs resulted in higher combined efficacy than with DOX, revealing that the choice of the antineoplastic has an important role in the success of the combination. These results also allow us to conclude that antimalarial drugs as repurposed drugs have enhanced effects in MCF-7 breast cancer cells, while combination with CNS drugs seems to be more effective in HT-29 colon cancer cells. These differences are visible when comparing the number of synergic pairs between the two cell lines and within the same line treated with different reference drugs (Table 2). Once again, this table contains results obtained previously in HT-29 and MCF-7 treated with the combination of antineoplastic drugs and antimalarials [28] to help the reader understand the drug pairs chosen in Section 3.2 for the evaluation of EMT biomarkers.

**Figure 4:**
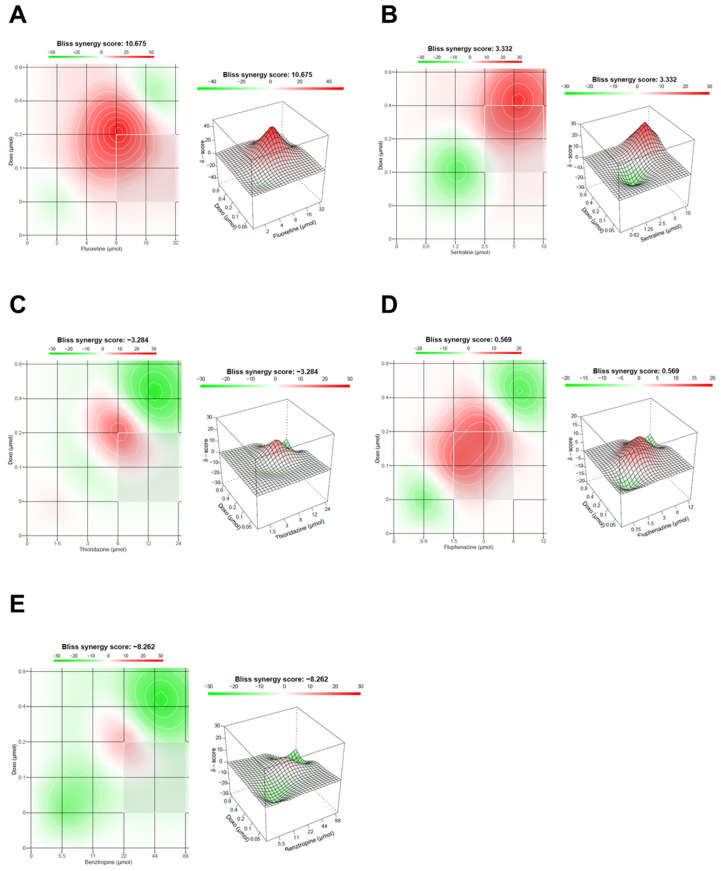
Bliss synergy plots of combined treatments of DOX and different CNS drugs on MCF-7 breast cancer cells. Bliss synergy plots of (**A**) DOX+fluoxetine, (**B**) DOX+sertraline, (**C**) DOX+thioridazine, (**D**) DOX+fluphenazine and (**E**) DOX+benztropine. The synergy score was calculated using SynergyFinder software. Positive or negative Bliss synergy scores indicate synergistic and antagonistic effects, respectively.

In addition to CompuSyn results, we also evaluate the drug interaction in these combinations with SynergyFinder, using the Bliss model. These results suggest that fluoxetine interacts most with DOX in intermediate concentrations, resulting in a synergy score of 10.675, indicative of synergism between the two drugs (Figure 4A). Sertraline combination with DOX also resulted in a positive Bliss score of 3.332, with synergic interactions with the reference drug for higher concentrations (Figure 4B). Both thioridazine and benztropine combinations with DOX resulted in negative scores of −3.284 and −8.262, respectively, indicative of antagonism (Figure 4C,E). Fluphenazine interaction with DOX gave a value of Bliss score near zero, indicating additivity (Figure 4D). These results demonstrate that using different reference models to evaluate the drug interactions between drug pairs can affect the synergism evaluation of these combinations. Nevertheless, these models usually result in similar results. 

### 3.2. Evaluation of EMT Biomarkers in MCF-7 and HT-29 Cells Treated with Repurposed Drugs Combined with Antineoplastic Agents

#### 3.2.1. EMT Biomarkers in MCF-7 Cells Treated with Antimalarial Drugs Combined with Antineoplastic Agents

After compiling all results obtained for HT-29 and MCF-7 cells treated with different combinations of repurposed drugs and antineoplastic agents, and after finding the most promising drug pairs for these cell lines, we evaluated if these drug combinations would affect the expression of some EMT biomarkers and if it was related to some degree of EMT reversion. Previously, in MCF-7 breast cancer cells, we have found that both artesunate and chloroquine resulted in significant cell viability reduction, especially when combined with DOX [28]. To evaluate EMT biomarkers expression, IHC studies were performed using the antibodies anti-E-cadherin, anti-P-cadherin, anti-β-catenin, and anti-vimentin. As our treatments resulted in a large decrease in the number of viable cells, we needed to adapt the traditional IHC protocol to minimize cell loss during the experiments. The traditional protocol of IHC using cells involves growing them in T-flasks, followed by trypsinization and centrifugation steps to obtain a pellet to embed in paraffin, creating cell blocks. Instead, we have seeded the cells in glass coverslips placed in 24-well plates, fixed and permeabilized them, and then performed the antigen retrieval, blocking, antibody incubations, and DAB reveal steps directly in each well. This allows avoiding morphological changes and cell losses due to the trypsinization and centrifugation steps needed for the cell blocks. After these steps, each coverslip was taken from each well with the help of forceps and was counterstained with hematoxylin, dehydrated, and permanently mounted in glass slides. This adapted protocol is also advantageous because it decreases the density of cells needed to obtain cell blocks and uses less volume of reagents. 

Results regarding E-cadherin expression (Figure S4) demonstrate that negative control does not show any labelling, confirming the success of the methodology. Single-drug treatments show similar expression of E-cadherin to the control cells (represented in the insert), except for chloroquine, whose treatment results in an increased expression of this protein. Results regarding the combination treatments DOX demonstrated higher expression of E-cadherin compared to control cells. There is also a smaller number of viable cells, with a pronounced change in morphology in the combined treatments. Chloroquine treatments also resulted in morphological changes at the nucleus.

The negative control of cells incubated with anti-P-cadherin does not show P-cadherin labelling, as expected. Similar labelling to the control cells (insert) is seen for all treatments both alone and in combination. In the combination of DOX + artesunate, there are some cells with a loss of expression of this protein (Figure S5).

Figure S6 represents the β-catenin labelling of MCF-7 cells treated with artesunate and chloroquine combined with DOX, to complement the previous results. For cells treated with each drug alone and combined, we found less protein labelling compared to control cells (insert), except for chloroquine. 

Finally, we also evaluated the labelling of vimentin, a biomarker of mesenchymal cells that is related to increased malignancy of cancer cells. We found no labelling of this protein in all treatments, except for cells treated with DOX alone, where slight labelling occurred (Figure S7). This may be due to the fact that some cells acquire resistance to this antineoplastic agent, becoming more aggressive.

#### 3.2.2. EMT Biomarkers in MCF-7 Cells Treated with CNS Drugs Combined with Antineoplastic Agents

We next evaluated the same EMT biomarkers in MCF-7 treated with another class of repurposed drugs (CNS agents), combined with PTX. Regarding E-cadherin, we found similar labelling of this protein compared to control cells (insert) in all single treatments. In combination with PTX, there is a more intense expression of E-cadherin, mainly in the combination of PTX with fluoxetine. There is also a smaller number of viable cells, with changes in morphology (Figure S8).

Next, we evaluated P-cadherin labelling in these cells and found a similarity between the control cells and the various treatments both alone and in combination (Figure S9). Treatment with fluoxetine and benztropine demonstrate some cells with loss of P-cadherin expression. 

Results regarding β-catenin labelling demonstrate less labelling than the control cells in the various treatments with each drug alone. In combination with PTX, there is a lower expression of protein labelling for all treatments compared to control cells, except in the combination of PTX and fluoxetine, whose labelling was similar to the control (Figure S10).

There is no labelling of vimentin protein in any treatment, both alone and combined, except for MCF-7 cells treated with benztropine, where slight labelling is shown. Interestingly, this marking is reversed when the cells are treated with benztropine in combination with PTX (Figure S11).

#### 3.2.3. EMT Biomarkers in HT-29 Cells Treated with CNS Drugs Combined with Antineoplastic Agents

Finally, these EMT biomarkers were also evaluated in HT-29 colon cancer cells treated with the most promising drug combinations using CNS agents. Similar E-cadherin labelling to the control cells is seen in the various treatments with each drug alone. There is more intense labelling in the combination treatments with 5-FU and all repurposed drugs. There is also a smaller number of viable cells with less aggregation ability, with a more pronounced change in morphology, especially in the combined treatments (Figure S12).

Regarding P-cadherin labelling, similar results were found between the control cells and 5-FU and sertraline treatments alone. There is an intense expression of P-cadherin in thioridazine treatments alone and combined with 5-FU, with lower intensity in the combination. There is also a smaller number of viable cells, especially in combination treatments, with smaller aggregates. In the combination of 5-FU with sertraline, it was found only a few viable cells (Figure S13).

There is a similar expression of β-catenin in the control cells and the treatment with 5-FU alone. Intense β-catenin labelling is seen in cells treated with thioridazine and sertraline alone; on the other hand, there is a reduction of the expression of this protein in the cells treated with these drugs combined with 5-FU. In all combination treatments, there is a notable change in cell morphology (Figure S14).

There is some labelling of vimentin protein in control cells and 5-FU alone, demonstrating a slight change in the characteristics of these cells, with some degree of transformation to a mesenchymal phenotype. This protein expression is reduced in all treatments with drugs both alone and combined (Figure S15).

## 4. Discussion

Our research group has been exploring the strategies of drug repurposing and drug combination as they represent a novel way to identify new potential candidates for cancer therapy. We make use of repurposed drugs, i.e., drugs already available on the market for other diseases than the original indication, and combine them with drugs already used in cancer therapy (antineoplastic agents) [46]. These strategies are advantageous as repurposed drugs have pharmacokinetics, pharmacodynamics, and toxicological profiles that are well established, making it easy for their approval for novel indications [47]. At the same time, if the combination with antineoplastic agents enhances their anticancer activity, it allows decreasing the therapeutical dose needed for the treatment and consequently the side effects [48]. We have been studying the repurposing of antimalarial and CNS drugs for breast and colon cancer and have already found promising drug pairs for colon and breast cancer. Previously, we have combined different antimalarial drugs with 5-FU for the colon and with DOX and PTX for breast cancer therapy [27,28]. Regarding the CNS drugs class, we have already tested the combination of these agents with 5-FU in HT-29 colon and with PTX in MCF-7 breast cancer cells. These studies aimed to evaluate if these drugs showed anticancer activity and mainly if their combination with antineoplastic drugs would enhance their cytotoxic effect on these cells. Collectively, we have found DOX+artesunate, DOX+chloroquine, PTX+fluoxetine, PTX+fluphenazine, and PTX+benztropine to be the most promising combinations for breast cancer therapy [27,28]. For colon cancer cells, we found 5-FU+thioridazine and 5-FU+sertraline to be the most effective drug pairs to reduce cell viability [27]. 

Here, and to complement our previous studies, we hypothesized that the combination of CNS drugs would also increase the antitumor potential of DOX in breast cancer cells and that this combination would be preferable to the combination with PTX. To do so, we treated MCF-7 breast cancer cells with different CNS drugs in increasing concentrations alone to find their IC_50_. Drugs that presented an IC_50_ under 25 µM were selected for combination with increasing concentrations of DOX. Combination efficacy was evaluated by MTT and SRB assays. Based on MTT results, analysis of drug interactions by calculation of CI was also performed to assess if CNS drugs acted synergically with DOX. The results for single treatments demonstrate that almost all CNS drugs, except for perampanel, have anticancer potential in MCF-7 cells. Regarding the combinations, we found some interesting drug pairs, such as DOX+fluoxetine, DOX+benztropine, and DOX+thioridazine, when combined in their IC_50_ values. Despite these results, compared to our previous findings, combination with PTX resulted in enhanced cell viability reduction compared to DOX, suggesting that these repurposed drugs should have some signalling pathway that may complement the mechanism of action of PTX but not DOX. These results also reinforce that the choice of the antineoplastic drug in different combination models with the same repurposed drugs can affect the results and should be performed very carefully. Following these results, CI calculation also demonstrated that most drug pairs presented an antagonistic or additive interaction, which supports our results from the viability studies. Collectively, these results also allow us to conclude that antimalarial drugs as repurposed drugs showed enhanced effects in MCF-7 breast cancer cells, while combination with CNS drugs seems to be more effective in HT-29 colon cancer cells.

Next, we compiled all results from our previous combination models with antimalarial and CNS drugs in HT-29 and MCF-7 cells and selected the most promising drug pairs to evaluate if these treatments resulted in changes in the expression of some EMT biomarkers such as E-cadherin, P-cadherin, vimentin, and β-catenin. The EMT is a process usually associated with cancer progression, where cells lose their epithelial features and acquire mesenchymal characteristics [49]. This phenomenon allows the cells to gain mobility and migrate from the primary tumour site, resulting in metastases. Different studies already suggested that EMT is linked to cancer progression [50,51,52,53,54,55]. TGF-β is involved in the regulation of cell proliferation and regulates the EMT by suppressing epithelial cells, leading to the loss of E-cadherin, an epithelial protein, while increasing the expression of mesenchymal biomarkers such as vimentin [56]. P-cadherin is another EMT biomarker that is usually co-expressed with E-cadherin and associated with poor prognosis, being associated with tumours with high metastatic potential [57]. On the other hand, β-catenin is usually overexpressed in more advanced staged cancers [58]. Vimentin is another protein that is overexpressed in mesenchymal cells and its increased expression is associated with malignancy and metastasis [59].

Generally, the findings presented in this manuscript demonstrate that the most promising combination treatments resulted in increased expression of E-cadherin and reduced expression of vimentin, β-catenin, and P-cadherin when compared to control cells, in both cell lines, which is indicative of a reversal in EMT, with cells expressing a typical profile of epithelial cells. Collectively, these results support that combination therapies can help reduce cell malignancy. 

Moreover, deeper mechanistic studies are strongly recommended to evaluate the anticancer mechanisms behind these drug combinations. These experiments should also be performed in other types of cancer, such as pancreatic, prostate, lung, etc., and further confirmed on animal models and clinal trials. Taken together, these results demonstrate that there is a great potential in the use of repurposed drugs and their inclusion in combination models for novel therapeutical strategies for colon and breast cancer.

## Figures and Tables

**Table 1:** IC_50_ of reference and repurposed drugs in MCF-7 breast and HT-29 colon cancer cells.

	Drug	HT-29 ^‡^	MCF-7
		IC_50_ (µM)	IC_50_ (µM)
	Doxorubicin	N.D.	0.17
	Paclitaxel		0.44 (nM)
	5-FU	3	N.D.
Central Nervous System	Rivastigmine	>100	N.D.
	m-chlorophenilbiguanide	>100	
	Safinamide	>100	
	Fluoxetine	6.12	7.78 *
	Benztropine	18.23	21.71 *
	Thioridazine	4.26	5.72 *
	Carbidopa	>100	N.D.
	Bromocriptine	>100	
	Nepicastat	61.24	
	Scopolamine	>100	
	Carbamazepine	>100	
	Sertraline	2.45	2.22 *
	Selegiline	>100	ND
	Entacapone	40.89	ND
	Tolcapone	35.47	ND
	Latrepirdine	7.75	75.37
	Fluphenazine	1.86	2.68 *
	Perampanel	>100	>100
Antimalarials ^‡^	Artesunate	17.88	11.60
	Chloroquine	32.13	N.D.
	Mefloquine	11.49	1.24
	6-methoxy-8-nitroquinoline	N.D.	>100
	Atovaquone		>100
	Cycloguanil		20.30
	Dapsone		53.99
	Ethosuximide		>100
	Lumefantrine		>100
	Natamycin		>100
	Piperazine		3.24
	Primaquine		29.90
	Primidone		>100
	Pyronaridine		1.39
	Quinidine		>100
	Rufinamide		>100
	Sitamaquine		>100
	Sulfamethoxazole		>100
	Tafenoquine		2.60
	Tobramycin		>100
	Tunicamycin		N.D.

* Selected for combination treatments with DOX; ^‡^ Based on our previous results [27,28]; N.D.—Not determined.

**Table 2:** Comparison of the number of synergic pairs in HT-29 and MCF-7 cell lines treated with the combination of CNS drugs (Drug B) and appropriate references (Drug A).

Drug A	Drug B	HT-29 *	MCF-7
		Number of Synergic Interactions	Number of Synergic Interactions
5-FU	Mefloquine	1	N.D.
	Artesunate	0	
	Latrepirdine	1	
	Fluphenazine	0	
	Fluoxetine	1	
	Benztropine	1	
	Thioridazine	3	
	Sertraline	5	
DOX	Artesunate	N.D.	4
	Chloroquine		3
	Mefloquine		1
	Pyronaridine		2
	Tafenoquine		0
	Fluoxetine		1
	Sertraline		0
	Thioridazine		0
	Benztropine		0
	Fluphenazine		0
PTX *	Artesunate	N.D.	2
	Chloroquine		2
	Mefloquine		1
	Pyronaridine		3
	Tafenoquine		0
	Fluoxetine		3
	Sertraline		2
	Thioridazine		3
	Benztropine		3
	Fluphenazine		3

* Based on our previous results [27,28]; N.D.: Not determined.

## Data Availability

The data presented in this study are contained within the manuscript.

## Supplementary Materials

The following supporting information can be downloaded at: https://www.mdpi.com/article/10.3390/biom12020190/s1, Figure S1: Detailed protocol of immunohistochemistry technique used in this work. Briefly, sterile 13 mm coverslips were placed in 24-well plates and coated with silane 2% in acetone for 1 h at room temperature. Before cell seeding, silane coverslips were washed with PBS and allowed to dry for 2 h. Next, cells were seeded and incubated for 24 h at 37 °C. After that period, cells were incubated with each treatment for 48 h. Cells were then washed with PBS and incubated with a solution of paraformaldehyde (PFA) 4% in PBS for 15 min at room temperature, for cell fixation. Next, cells were permeabilized with PBS-0.1% Triton X-100. Antigen retrieval (unmasking) was performed using a Retrieval Solution (10% in water). The retrieval solution was boiled and 500 µL were transferred to each well, followed by incubation at 60 °C for 30 min. After that, cells were washed twice in triphosphate buffered saline (TBS) for 5 min. Endogenous peroxidase was blocked using 5 drops per well of Peroxidase Block Solution for 5 min at room temperature and washed twice in TBS for 5 min. Next, protein block was performed using 5 drops per well of Protein Block solution for 1 h at room temperature. Slips were then washed twice in TBS for 5 min. Afterwards, the coverslips were incubated with agitation overnight, at 4 °C with 200 µL of each primary antibody. Next, cells were washed twice in TBS, for 5 min and 4 drops of the Post Primary solution was added to each well, following an incubation time of 30 min at room temperature. Once again, the slips were washed twice in TBS, for 5 min. After this time, they were incubated with 5 drops of Novolink Polymer per well for 30 min and then washed twice in TBS for 5 min. Next, the peroxidase activity was developed by adding 200 μL of a solution of DAB to each well. Finally, each coverslip was removed from the 24-well plate using a small pair of broad-tipped forceps and rinsed in water, counterstained in haematoxylin for 30 s, washed again for 5 to 10 min, dehydrated, diaphanized in xylene, and the slides were mounted. Figure S2: Microscopic cellular visualisation of MCF-7 cells after treatment with the combination of different CNS drugs and DOX. Cells treated with vehicle (0.1% DMSO) were used as control. Scale bar: 50 µM. Figure S3: Linear regression analysis of the results obtained by MTT and SRB assays. The effect of (A) DOX + fluoxetine (B) DOX + sertraline (C) DOX + thioridazine (D) DOX + fluphenazine and (E) DOX + benztropine on cell viability. Each point represents the mean ± SD relative to the control cells. * Statistically significant from MTT assay at *p* < 0.05. ** statistically significant from MTT assay at *p* < 0.01. *** statistically significant from MTT assay at *p* < 0.001. **** statistically significant from MTT assay at *p* < 0.0001. Figure S4: Expression of E-cadherin in MCF-7 cells, by IHC. Treatments are described in the upper left corner of each image. All images were obtained at a magnification of 400×. Insert represents control cells (treated with 0.1% DMSO). CTR -: negative control. Figure S5: Expression of P-cadherin in MCF-7 cells, by IHC. Treatments are described in the upper left corner of each image. All images were obtained at a magnification of 400×. Insert represents control cells (treated with 0.1% DMSO). CTR -: negative control. Figure S6: Expression of β-catenin in MCF-7 cells, by IHC. Treatments are described in the upper left corner of each image. All images were obtained at a magnification of 400×. Insert represents control cells (treated with 0.1% DMSO). CTR -: negative control. Figure S7: Expression of vimentin in MCF-7 cells, by IHC. Treatments are described in the upper left corner of each image. All images were obtained at a magnification of 400×. Insert represents control cells (treated with 0.1% DMSO). CTR -: negative control. Figure S8: Expression of E-cadherin in MCF-7 cells, by IHC. Treatments are described in the upper left corner of each image. All images were obtained at a magnification of 400×. Insert represents control cells (treated with 0.1% DMSO). CTR -: negative control. Figure S9: Expression of P-cadherin in MCF-7 cells, by IHC. Treatments are described in the upper left corner of each image. All images were obtained at a magnification of 400×. Insert represents control cells (treated with 0.1% DMSO). CTR -: negative control. Figure S10: Expression of β-catenin in MCF-7 cells, by IHC. Treatments are described in the upper left corner of each image. All images were obtained at a magnification of 400×. Insert represents control cells (treated with 0.1% DMSO). CTR -: negative control. Figure S11: Expression of vimentin in MCF-7 cells, by IHC. Treatments are described in the upper left corner of each image. All images were obtained at a magnification of 400×. Insert represents control cells (treated with 0.1% DMSO). CTR -: negative control. Figure S12: Expression of E-cadherin in HT-29 cells, by IHC. Treatments are described in the upper left corner of each image. All images were obtained at a magnification of 400×. Insert represents control cells (treated with 0.1% DMSO). CTR -: negative control. Figure S13: Expression of P-cadherin in HT-29 cells, by IHC. Treatments are described in the upper left corner of each image. All images were obtained at a magnification of 400×. Insert represents control cells (treated with 0.1% DMSO). CTR -: negative control. Figure S14: Expression of β-catenin in HT-29 cells, by IHC. Treatments are described in the upper left corner of each image. All images were obtained at a magnification of 400×. Insert represents control cells (treated with 0.1% DMSO). CTR -: negative control. Figure S15: Expression of vimentin in HT-29 cells, by IHC. Treatments are described in the upper left corner of each image. All images were obtained at a magnification of 400×. Insert represents control cells (treated with 0.1% DMSO). CTR -: negative control.
