# Supplementary material for: Drug Combinations: A New Strategy to Extend Drug Repurposing and Epithelial-Mesenchymal Transition in Breast and Colon Cancer Cells
Source: Biomolecules. 2022 Jan 23;12(2):190. doi: 10.3390/biom12020190 (PMC8961626; doi:10.3390/biom12020190)
Supplement: Supplementary file 1 [file biomolecules-12-00190-s001.zip › biomolecules-1528902-supplementary.pdf]

Drug combinations: a new strategy to extend drug repurposing and epithelial-mesenchymal transition in breast and colon cancer cells

Diana Duarte [1,2], Alexandra Rêma [3], Irina Amorim [3,4,5] and Nuno Vale [1,6,*]

1 OncoPharma Research Group, Center for Health Technology and Services Research (CINTESIS), Rua Doutor Plácido da Costa, 4200-450 Porto, Portugal
2 Faculty of Pharmacy of University of Porto, Rua Jorge Viterbo Ferreira, 228, 4050-313 Porto, Portugal
3 Department of Pathology and Molecular Immunology, Institute of Biomedical Sciences Abel Salazar (ICBAS), University of Porto, 4050-313 Porto, Portugal
4 Institute of Pathology and Molecular Immunology of the University of Porto (IPATIMUP), 4200-465 Porto, Portugal
5 i3S-Instituto de Investigação e Inovação em Saúde, Universidade do Porto, 4200-135 Porto, Portugal
6 Department of Community Medicine, Health Information and Decision (MEDCIDS), Faculty of Medicine, University of Porto, Alameda Professor Hernâni Monteiro, 4200-319 Porto, Portugal

Supplementary Materials

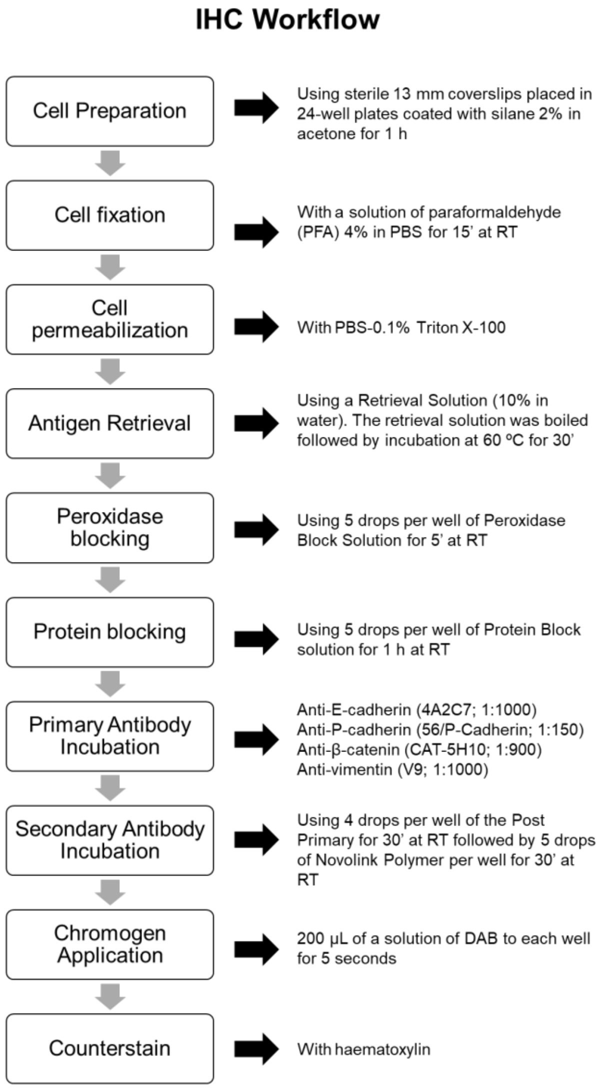

Figure S1. Detailed protocol of immunohistochemistry technique used in this work. Briefly, sterile 13 mm coverslips were placed in 24-well plates and coated with silane 2% in acetone for 1 h at room temperature. Before cell seeding, silane coverslips were washed with PBS and allowed to dry for 2 h. Next, cells were seeded and incubated for 24 h at 37 °C. After that period, cells were incubated with each treatment for 48 h. Cells were then washed with PBS and incubated with a solution of paraformaldehyde (PFA) 4% in PBS for 15 min at room temperature, for cell fixation. Next, cells were permeabilized with PBS-0.1% Triton X-100. Antigen retrieval (unmasking) was performed using a Retrieval Solution (10% in water). The retrieval solution was boiled and 500 μL were transferred to each well, followed by incubation at 60 °C for 30 min. After that, cells were washed twice in triphosphate buffered saline (TBS) for 5 min. Endogenous peroxidase was blocked using 5 drops per well of Peroxidase Block Solution for 5 min at room temperature and washed twice in TBS for 5 min. Next, protein block was performed using 5 drops per well of Protein Block solution for 1 h at room temperature. Slips were then washed twice in TBS for 5 min. Afterwards, the coverslips were incubated with agitation overnight, at 4 °C with 200 μL of each primary antibody. Next, cells were washed twice in TBS, for 5 min and 4 drops of the Post Primary solution was added to each well, following an incubation time of 30 min at room temperature. Once again, the slips were washed twice in TBS, for 5 min. After this time, they were incubated with 5 drops of Novolink Polymer per well for 30 min and then washed twice in TBS for 5 min. Next, the peroxidase activity was developed by adding 200 μL of a solution of DAB to each well. Finally, each coverslip was removed from the 24-well plate using a small pair of broad-tipped forceps and rinsed in water, counterstained in haematoxylin for 30 sec, washed again for 5 to 10 min, dehydrated, diaphanized in xylene, and the slides were mounted.

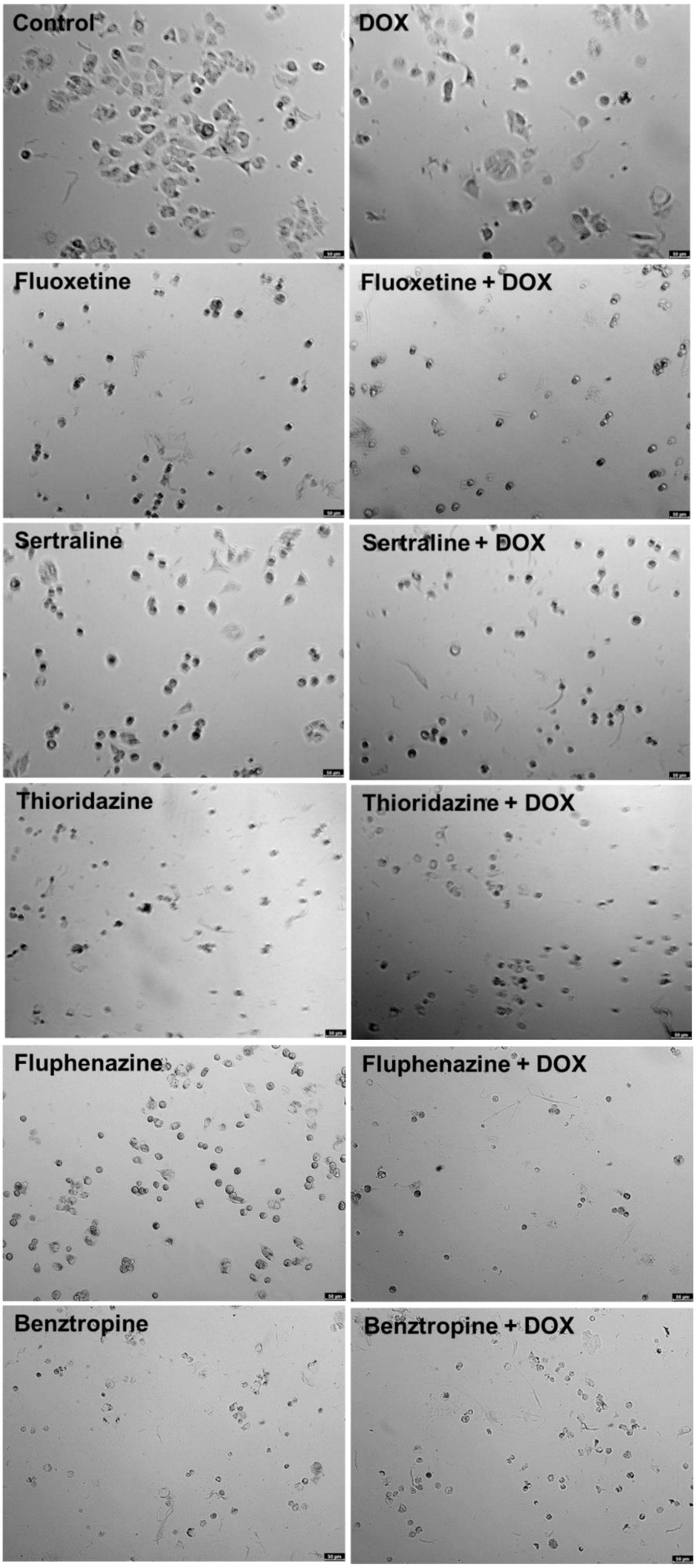

Figure S2. Microscopic cellular visualisation of MCF-7 cells after treatment with the combination of different CNS drugs and DOX. Cells treated with vehicle (0.1% DMSO) were used as control. Scale bar: 50 μM.

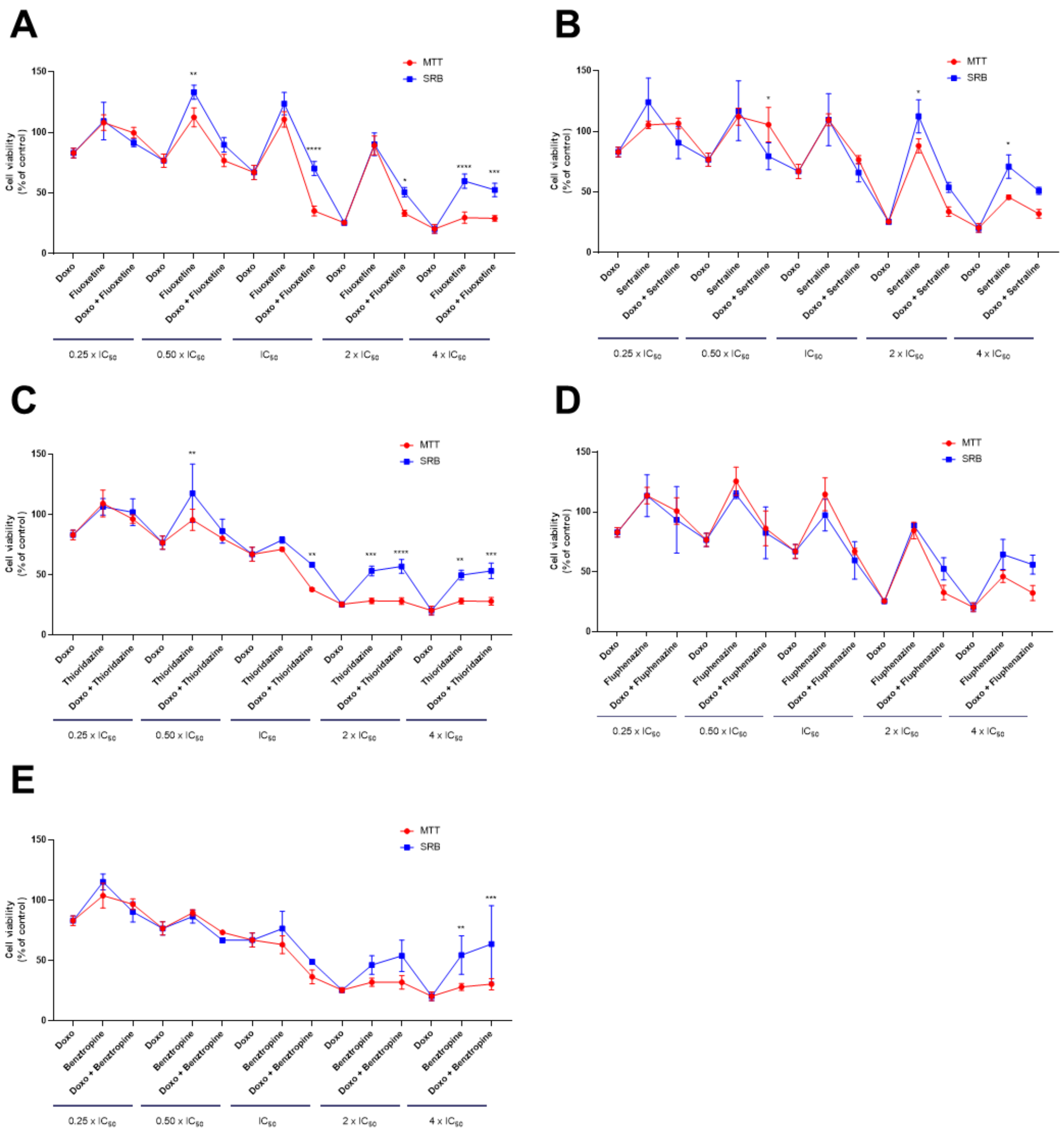

Figure S3. Linear regression analysis of the results obtained by MTT and SRB assays. The effect of (A) DOX + fluoxetine (B) DOX + sertraline (C) DOX + thioridazine (D) DOX + fluphenazine and (E) DOX + benztropine on cell viability. Each point represents the mean ± SD relative to the control cells. * Statistically significant from MTT assay at $p < 0.05$. ** statistically significant from MTT assay at $p < 0.01$. *** statistically significant from MTT assay at $p < 0.001$. **** statistically significant from MTT assay at $p < 0.0001$.

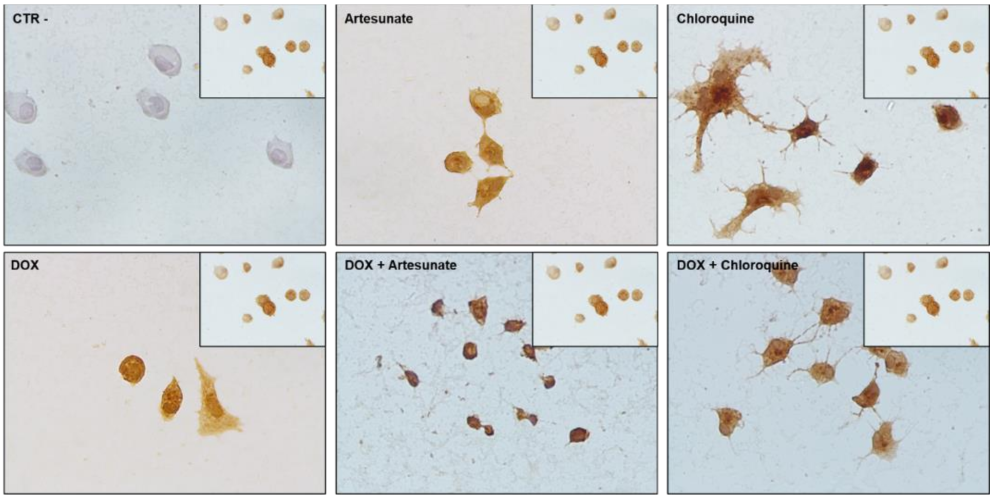

Figure S4. Expression of E-cadherin in MCF-7 cells, by IHC. Treatments are described in the upper left corner of each image. All images were obtained at a magnification of 400x. Insert represents control cells (treated with 0.1% DMSO). CTR -: negative control.

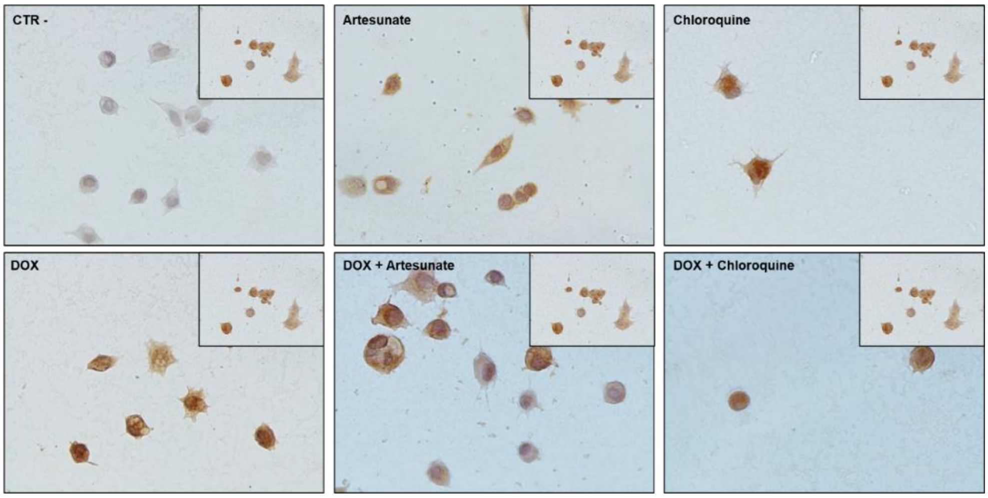

Figure S5. Expression of P-cadherin in MCF-7 cells, by IHC. Treatments are described in the upper left corner of each image. All images were obtained at a magnification of 400x. Insert represents control cells (treated with 0.1% DMSO). CTR -: negative control.

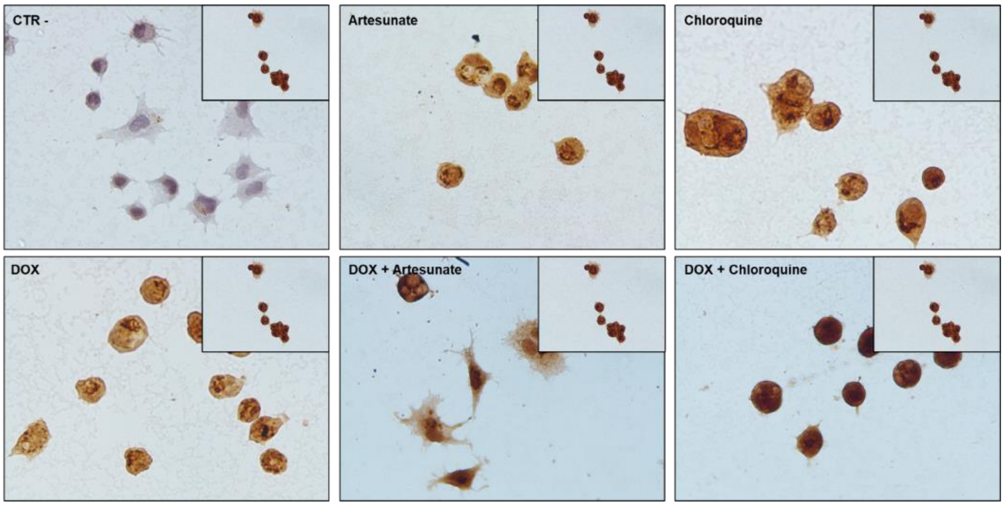

Figure S6. Expression of β-catenin in MCF-7 cells, by IHC. Treatments are described in the upper left corner of each image. All images were obtained at a magnification of 400x. Insert represents control cells (treated with 0.1% DMSO). CTR -: negative control.

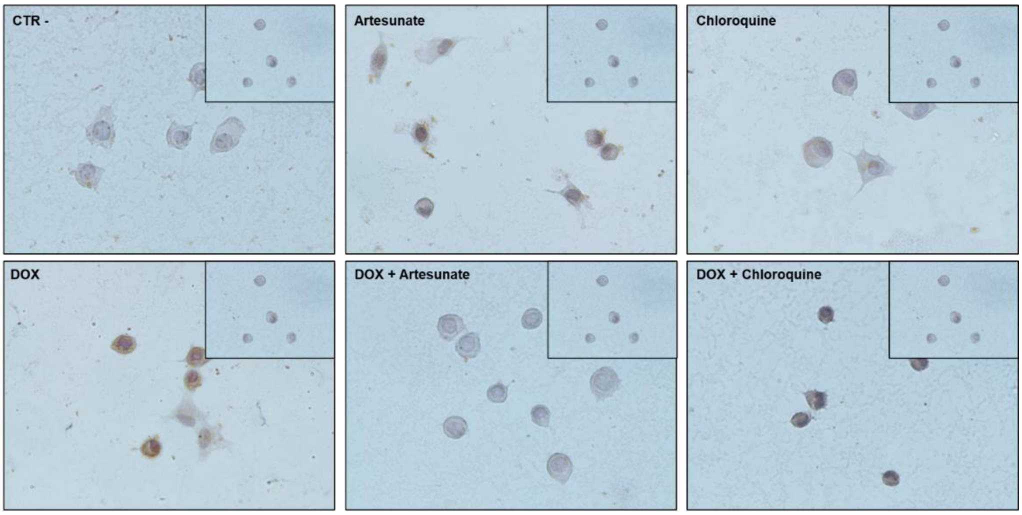

Figure S7. Expression of vimentin in MCF-7 cells, by IHC. Treatments are described in the upper left corner of each image. All images were obtained at a magnification of 400x. Insert represents control cells (treated with 0.1% DMSO). CTR -: negative control.

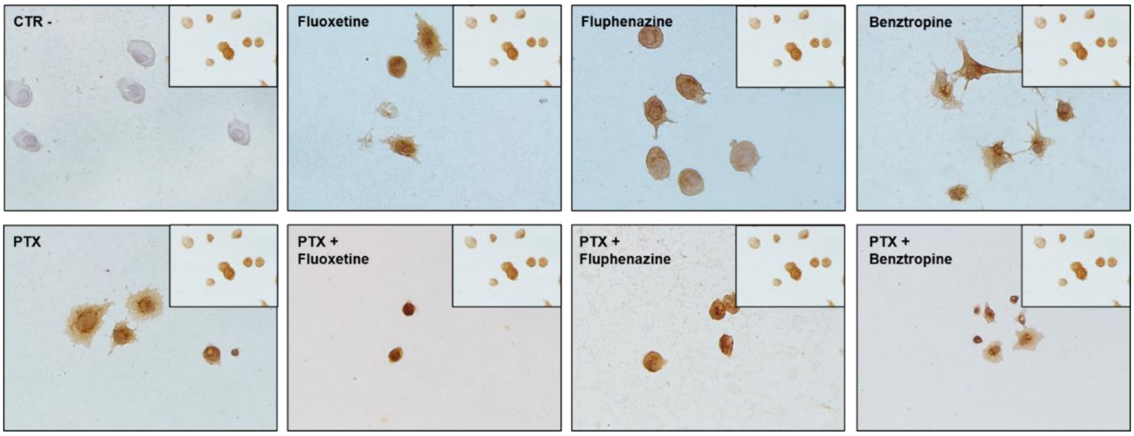

Figure S8. Expression of E-cadherin in MCF-7 cells, by IHC. Treatments are described in the upper left corner of each image. All images were obtained at a magnification of 400x. Insert represents control cells (treated with 0.1% DMSO). CTR -: negative control.

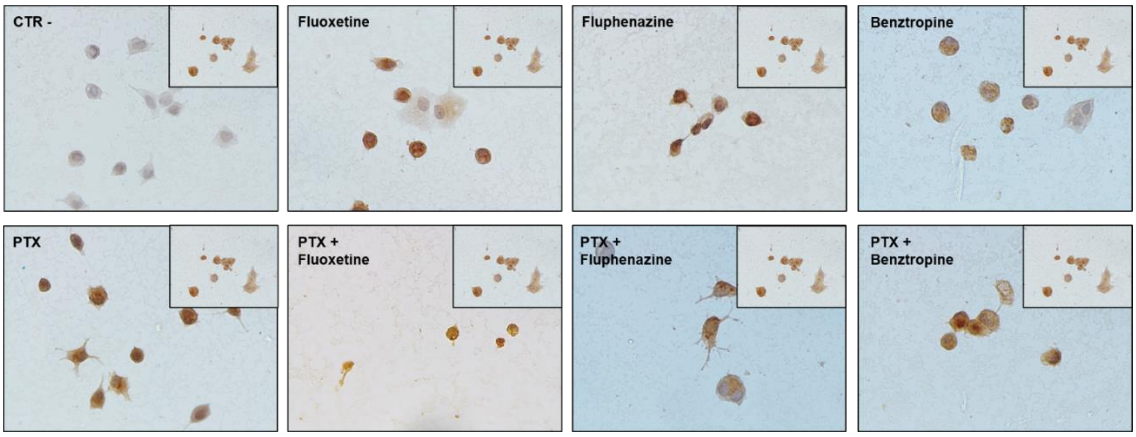

Figure S9. Expression of P-cadherin in MCF-7 cells, by IHC. Treatments are described in the upper left corner of each image. All images were obtained at a magnification of 400x. Insert represents control cells (treated with 0.1% DMSO). CTR -: negative control.

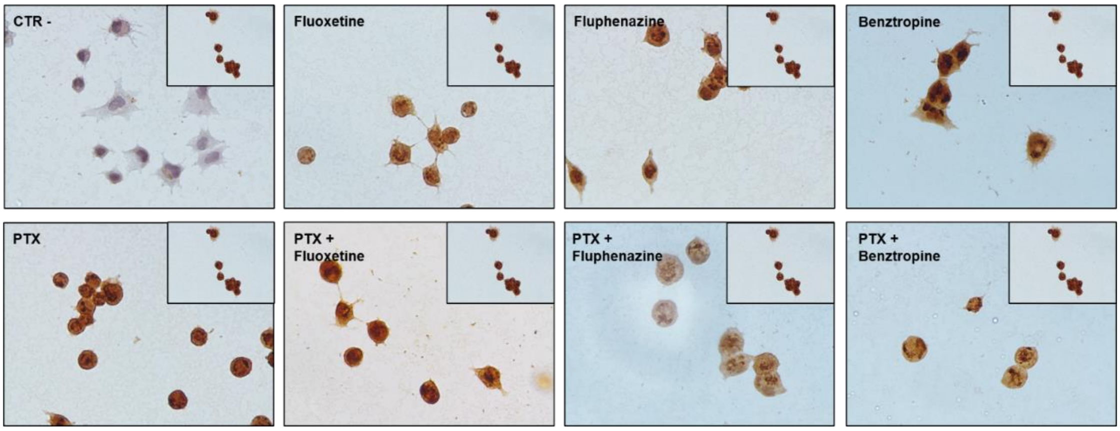

Figure S10. Expression of β-catenin in MCF-7 cells, by IHC. Treatments are described in the upper left corner of each image. All images were obtained at a magnification of 400x. Insert represents control cells (treated with 0.1% DMSO). CTR -: negative control.

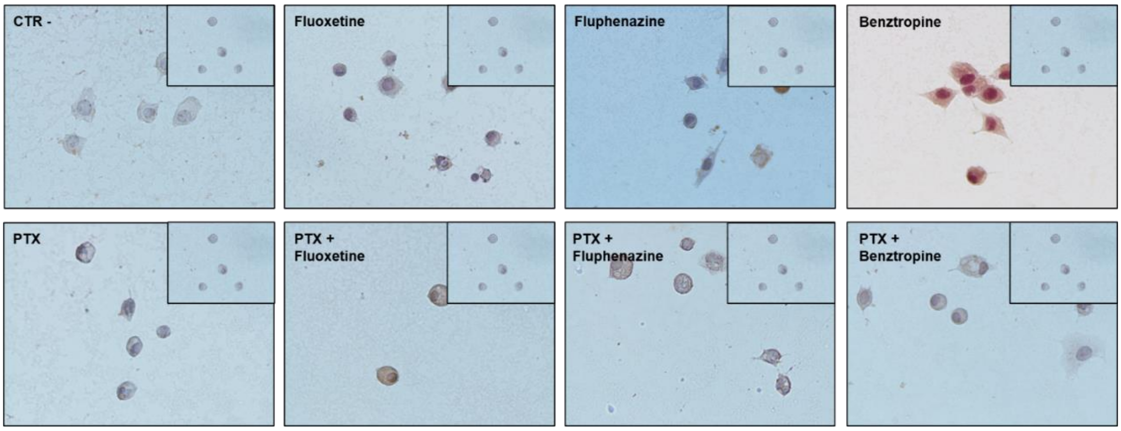

Figure S11. Expression of vimentin in MCF-7 cells, by IHC. Treatments are described in the upper left corner of each image. All images were obtained at a magnification of 400x. Insert represents control cells (treated with 0.1% DMSO). CTR -: negative control.

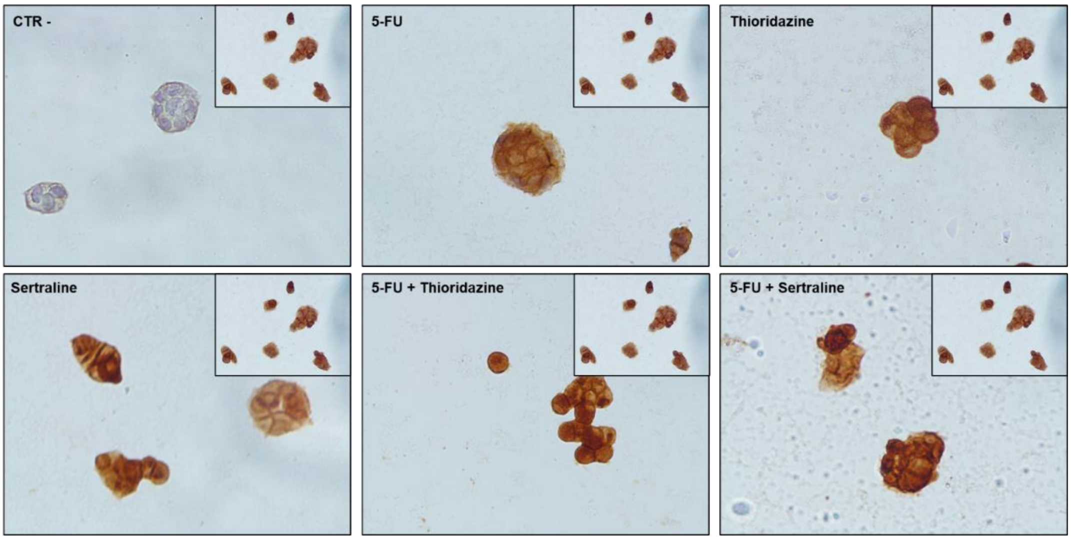

Figure S12. Expression of E-cadherin in HT-29 cells, by IHC. Treatments are described in the upper left corner of each image. All images were obtained at a magnification of 400x. Insert represents control cells (treated with 0.1% DMSO). CTR -: negative control.

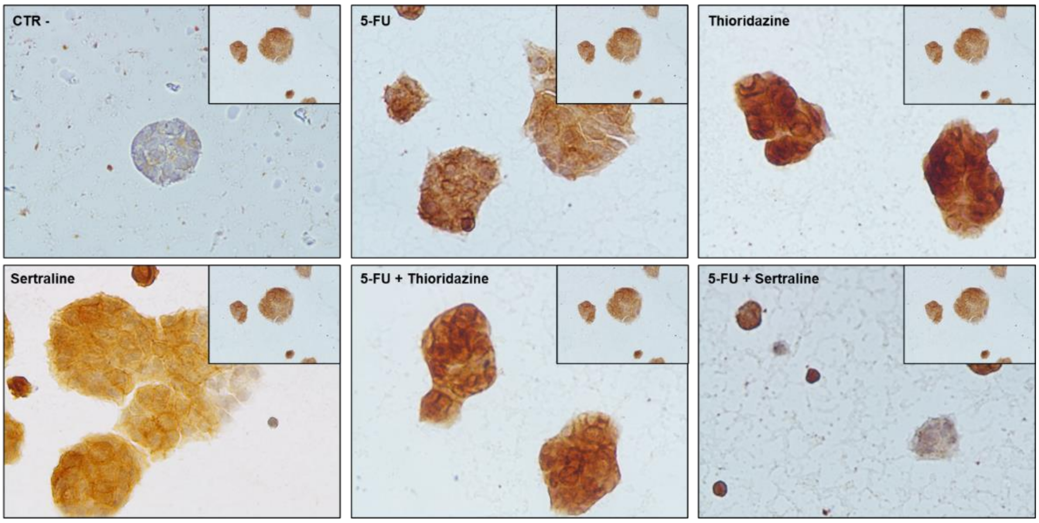

Figure S13. Expression of P-cadherin in HT-29 cells, by IHC. Treatments are described in the upper left corner of each image. All images were obtained at a magnification of 400x. Insert represents control cells (treated with 0.1% DMSO). CTR -: negative control.

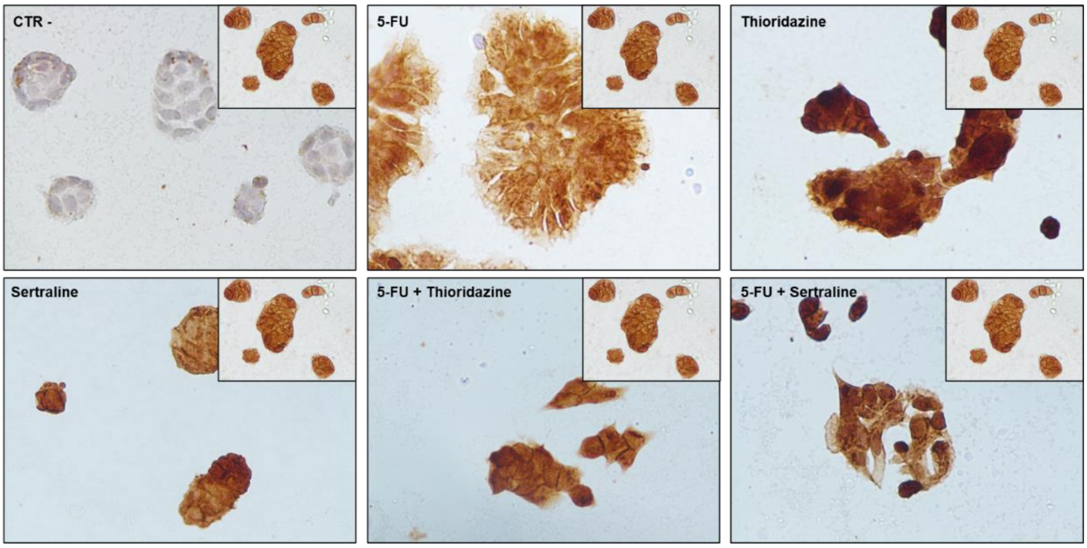

Figure S14. Expression of β-catenin in HT-29 cells, by IHC. Treatments are described in the upper left corner of each image. All images were obtained at a magnification of 400x. Insert represents control cells (treated with 0.1% DMSO). CTR -: negative control.

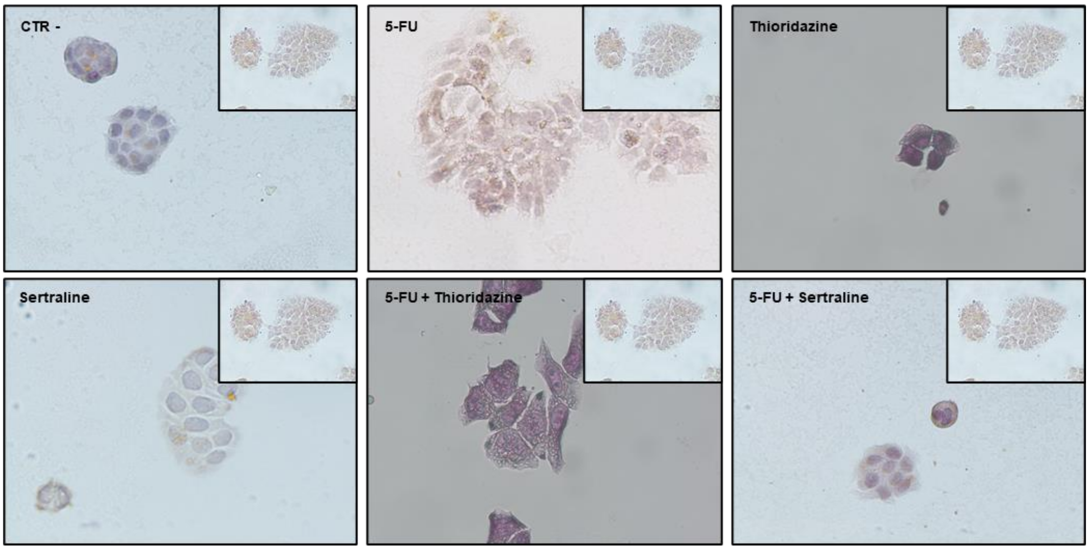

Figure S15. Expression of vimentin in HT-29 cells, by IHC. Treatments are described in the upper left corner of each image. All images were obtained at a magnification of 400x. Insert represents control cells (treated with 0.1% DMSO). CTR -: negative control.
